# Supplementary material for: Hibiscus Flower and Olive Leaf Extracts Activate Apoptosis in SH-SY5Y Cells
Source: Antioxidants (Basel). 2021 Dec 7;10(12):1962. doi: 10.3390/antiox10121962 (PMC8750347; doi:10.3390/antiox10121962)
Supplement: Supplementary file 1 [file antioxidants-10-01962-s001.zip › antioxidants-1472460-supplementary.pdf]

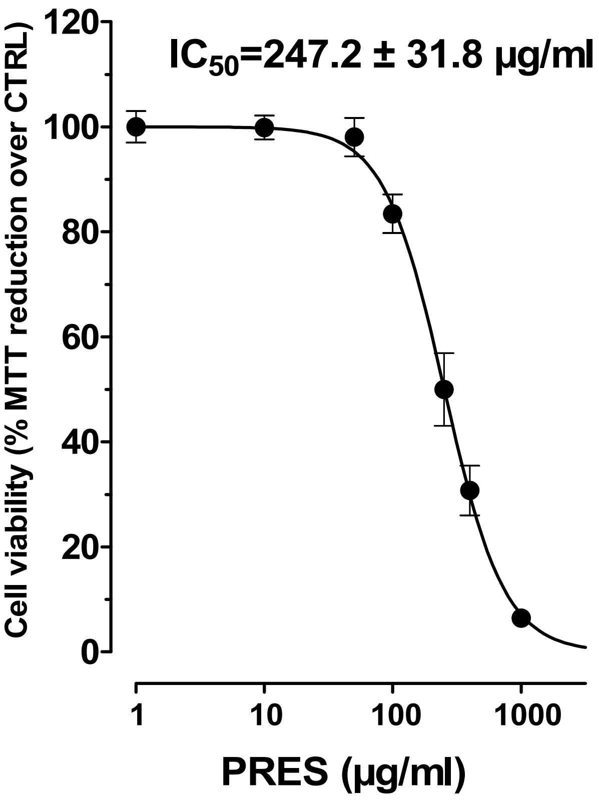

Figure S1. Graphical determination of PRES IC50 obtained by fitting data according to a sigmoid equation with variable slope. IC50 is reported as mean ± SEMs of six independent experiments.
